# Trivial Indirect Trauma Causing Recurrent Bilateral Quadriceps Tendon Rupture in End Stage Renal Failure Patient: A Case Report

**DOI:** 10.5704/MOJ.2111.021

**Published:** 2021-11

**Authors:** NH Fakru, D Ruslan, M Tengku

**Affiliations:** 1 Department of Orthopaedics, Universiti Sains Malaysia, Kubang Kerian, Malaysia; 2 Department of Orthopaedics, Hospital Raja Permaisuri Bainun, Ipoh, Malaysia

**Keywords:** recurrent, bilateral, quadriceps tendon rupture

## Abstract

Recurrent bilateral quadriceps tendon rupture in a young patient is a very rare incident. The underlying medical condition is always present and may have contributed to this injury. We report a recurrent bilateral quadricep tendon rupture in a 28-year-old man with underlying end-stage renal failure that occurred 10 months after the first repair. Injuries were indirect and trivial during the first and second events. Surgical repair was performed with similar technique for both incidents and he was advised to exercise extreme cautions after the second repair. He could return to his daily activities with no further recurrence at 30 months follow-up.

## Introduction

Quadriceps tendon rupture (QTR) is a well-known injury as a result of direct trauma. However, bilateral QTRs following an indirect and minor injury is very rare, with the elderly being at higher risk and the underlying medical conditions are attributable^1^.

We present a rare case that involved a young patient with a recurrent simultaneous rupture of bilateral quadriceps tendon caused by a trivial injury.

## Case Report

A 28-year-old Malay gentleman with underlying end-stage renal failure (ESRF) and hypertension, presented with history of slipped and fell while walking down the stairs. He had been on regular hemodialysis for the three years. He was not a smoker or an alcoholic. Post-trauma, he complained of bilateral knee pain and swelling, and was unable to ambulate. On examination, swelling and tenderness were noted over bilateral superior patella regions with knee extensor mechanism deficit. His Body Mass Index (BMI) was eighteen. No fracture was identified on plain radiographs. Ultrasonography confirmed bilateral QTR diagnosis, and bilateral repair was performed. Intra-operatively, fibrous tissue was noted at bilateral suprapatellar region. Both tendons were repaired with Krackow suture technique. The tendons were fixed to patella bone through multiple tunnels drilled across the patellae using braided non-absorbable Etibond size 5.0 suture, four strands over right knee and six strands over left knee. Both knees were protected with posterior slab for five weeks. As for rehabilitation, we modified the Rosenberg Cooley Metcalf post-operative protocol for quadricep tendon repair. Two months post-operatively, he was allowed full weight bearing with bilateral knee range of motion (ROM) of 0° to 100° and he resumed his daily activities thereafter. No specific investigation was done to confirm the tendon healing.

**Fig. 1:**
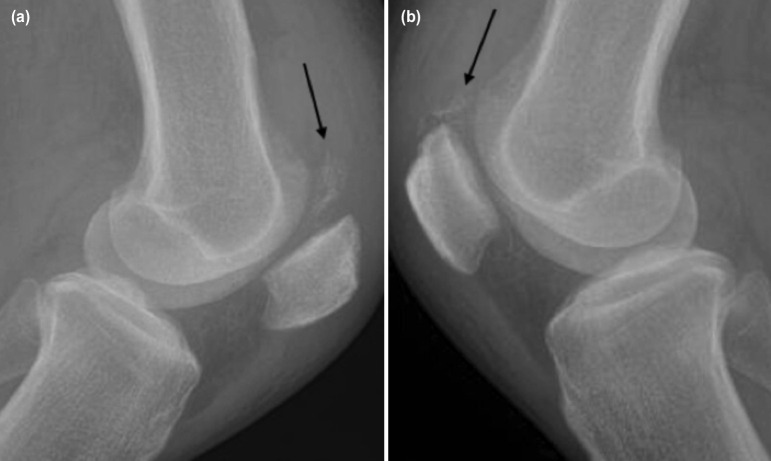
Lateral plain radiograph of the knee shows calcification (arrow) at the quadricep tendon insertion with patella baha, (a) right knee, (b) left knee.

**Fig. 2:**
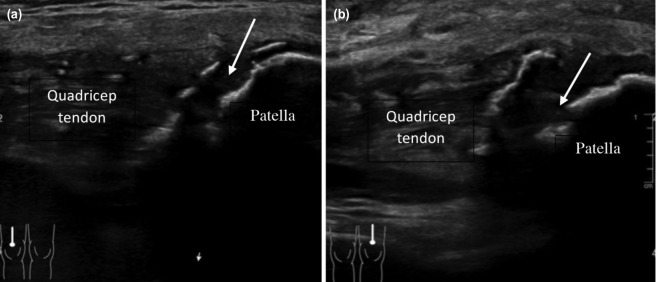
Ultrasound of bilateral knee shows the gap (arrow) in the quadricep tendon which indicates re-rupture, (a) right knee, (b) left knee.

However, he presented again ten months later with complaints of inability to ambulate associated with bilateral knee pain and swelling after a trivial fall. A ‘pop’ sound was heard during the fall. Clinical assessment showed loss of bilateral knee extensor mechanism. The plain radiograph showed bilateral patella baja (Fig. 1) and ultrasonography confirmed the diagnosis of re-rupture of bilateral quadriceps tendon (Fig. 2). His serum iPTH was 381 pmol/l, which was high, indicating hyperparathyroidism.

**Fig. 3:**
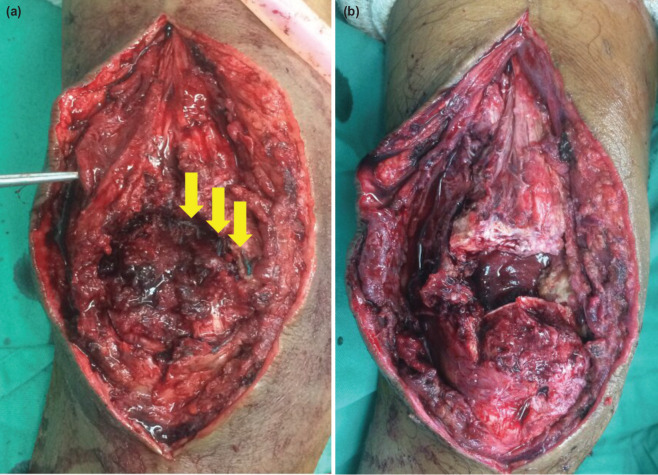
Shows intra-operative findings of recurrent rupture of right knee. (a) Rupture at tendon-bone interface with disrupt suture material (arrows), (b) appearance after clearing the hematoma and disrupted sutures.

Surgery was performed and intra-operatively, it was noted that the ruptures occurred at the previous repaired site at the tendon-bone junction. Fibrosis was noted at the bilateral quadriceps tendon stumps with suture rupture from previous surgery (Figure 3). After the fibrosis was debrided, direct tendon-to-bone repair was done. Six strands of braided non-absorbable size 5 sutures were used to anchor the tendon directly on the patella via five tunnels drilled vertically across the patella. Krackow suture technique was used to fix the tendon and both knees were protected with posterior slab post-operatively for seven weeks. Similar to first surgery, no biological substance was used to enhance the healing. The same rehabilitation protocol was used but we further delayed the knee range of motion in which we achieved 90° flexion at eighth week and full weight bearing at nine weeks.

Five months after surgery, the patient resumed his normal daily activities. No investigation was done to confirm the tendon healing. His knees range of motion were 0° to 130° at one-year follow-up. His hyperparathyroidism was managed by the general surgical team where he subsequently underwent parathyroidectomy. The quadriceps tendons remained intact during the last visit at 30 months after the second repair.

## Discussion

Recurrent bilateral QTR resulting from indirect trauma is very rare. Literature review by authors found only one similar case report. Sherman *et al* highlighted this incident in a 58-year-old man with sickle cell disease in 20171. His patient had risk factors such as a long previous history of bilateral quadriceps tendon rupture, hypertension, tobacco exposure and chronic alcohol consumption^1^. The author also highlighted various other potential risk factors that may predispose to QTR such as diabetes, hypertension, gout, polyneuropathy, osteogenesis imperfecta, collagen diseases, rheumatic arthritis and calcific tendinosis. Patient’s factors such as chronic alcohol consumption, smoking, obesity, and anatomical variant could also contribute. Additionally, statins and steroids also play a role^1^.

Our case involved a young 28-year-old man who had hyperparathyroidism and end-stage renal failure (ESRF) with three years dependency on hemodialysis. He was ten months post QTR repair when the recurrent rupture occurred. Although there are many ESRF patients on dialysis worldwide, to our knowledge, the incidence of recurrent simultaneous bilateral QTR has never been reported so far. This could be attributed to relatively older ESRF patients who would be less physically active. On the contrary, our patient was young and active, posing higher risk of tendon rupture and recurrence. Additionally, no post-operative investigation to evaluate tendon healing was done. In an uncomplicated case, this assessment is not a routine but should be highly recommended in a complicated case to avoid re-rupture.

Several ESRF-related sequalae are thought to be related to tendon weakening. Previous studies suggest that spontaneous tendon rupture is related to the duration of hemodialysis^2^. Additionally, hyperparathyroidism is postulated to have increased osteoclastic activity. Chronically elevated PTH leads to increased subperiosteal bone resorption, resulting in weakening of tendon-osseous junction^3^. Eventually, rupture can occur with low-impact indirect injury. Another possible mechanism of tendon rupture in hyperparathyroidism is increased bone collagenase activity which accelerates insoluble collagen degradation, leading to tendon weakening^3^. It was also suggested that accumulation of uremic toxins, chronic acidosis and amyloidosis may contribute to tendon rupture in ESRF^4^.

In 2013, McCarron *et al* showed that tendon retraction without defect is a normal phenomenon if primary repair is inadequately protected. The term ‘failure with continuity’ was suggested when bone-tendon integration was not achieved despite bone-tendon-muscle continuity^5^. Therefore, evaluating post repair with specific investigation would be helpful to ensure appropriate healing has occur. We believe our second repair survived could also be due to slower rehabilitation that result in ‘adequately protected’ tendon.

Patients with complicated QTR require meticulous repair with possible biological augmentation, as well as a conservative rehabilitation protocol and rigorous follow-up, to ensure healing has occurred before returning to full activities. A specific investigation to assess the tendon healing together with appropriate rehabilitation protocol would be necessary to ensure good outcome.
